# Real-Time fMRI Neurofeedback Training of Amygdala Activity in Patients with Major Depressive Disorder

**DOI:** 10.1371/journal.pone.0088785

**Published:** 2014-02-11

**Authors:** Kymberly D. Young, Vadim Zotev, Raquel Phillips, Masaya Misaki, Han Yuan, Wayne C. Drevets, Jerzy Bodurka

**Affiliations:** 1 Laureate Institute for Brain Research, Tulsa, Oklahoma, United States of America; 2 Janssen Pharmaceuticals, LCC, of Johnson & Johnson, Inc., Titusville, New Jersey, United States of America; 3 Center for Biomedical Engineering, The University of Oklahoma, Norman, Oklahoma, United States of America; 4 College of Engineering, The University of Oklahoma, Norman, Oklahoma, United States of America; Bellvitge Biomedical Research Institute-IDIBELL, Spain

## Abstract

**Background:**

Amygdala hemodynamic responses to *positive* stimuli are *attenuated* in major depressive disorder (MDD), and normalize with remission. Real-time functional MRI neurofeedback (rtfMRI-nf) offers a non-invasive method to modulate this regional activity. We examined whether depressed participants can use rtfMRI-nf to enhance amygdala responses to positive autobiographical memories, and whether this ability alters symptom severity.

**Methods:**

Unmedicated MDD subjects were assigned to receive rtfMRI-nf from either left amygdala (LA; experimental group, n = 14) or the horizontal segment of the intraparietal sulcus (HIPS; control group, n = 7) and instructed to contemplate happy autobiographical memories (AMs) to raise the level of a bar representing the hemodynamic signal from the target region to a target level. This 40s Happy condition alternated with 40s blocks of rest and counting backwards. A final Transfer run without neurofeedback information was included.

**Results:**

Participants in the experimental group upregulated their amygdala responses during positive AM recall. Significant pre-post scan decreases in anxiety ratings and increases in happiness ratings were evident in the experimental versus control group. A whole brain analysis showed that during the transfer run, participants in the experimental group had increased activity compared to the control group in left superior temporal gyrus and temporal polar cortex, and right thalamus.

**Conclusions:**

Using rtfMRI-nf from the left amygdala during recall of positive AMs, depressed subjects were able to self-regulate their amygdala response, resulting in improved mood. Results from this proof-of-concept study suggest that rtfMRI-nf training with positive AM recall holds potential as a novel therapeutic approach in the treatment of depression.

## Introduction

Major Depressive Disorder (MDD) is a disabling and common medical condition[1]. Approximately two-thirds of patients who seek pharmacological and/or psychological interventions will not respond fully to treatment, and only one-half of treatment-responders achieve sustained remission[2]. Cognitive-behavioral therapy (CBT), the most commonly implemented psychological treatment for MDD, is most effective for mildly-to-moderately depressed patients[3], but is generally insufficient as monotherapy for severely ill patients[4]. Treatments available for severely ill patients who don't respond to multiple conventional treatments such as psychotherapy, pharmacotherapy, and/or a combination of the two include electroconvulsive therapy, vagus nerve stimulation, and deep brain stimulation, which are invasive and associated with significant adverse event risks[5], [6]. Therefore, substantial need exists to investigate novel therapeutic approaches for MDD that can improve the effectiveness of non-invasive treatments.

Real-time functional magnetic resonance imaging (rtfMRI), in which blood oxygen-level-dependent (BOLD) fMRI data processing and display are performed concomitantly with image acquisition[7], has enabled rtfMRI neurofeedback (rtfMRI-nf) training, allowing a person to see and regulate the fMRI signal from their own brain[8]. Contrary to other biofeedback methods (such as EEG), rtfMRI-nf training results in the precise localization and modulation of relevant brain structures, allowing focal investigation of relationships between cognitive-behavioral functions and neuroplasticity changes[9], [10]. By using rtfMRI-nf, healthy individuals can learn to self-regulate brain activity in structures relevant to emotional processing including the insula, amygdala, ventrolateral prefrontal cortex (VLPFC), and anterior cingulate cortex (ACC)[11]–[15]. Emerging evidence also suggests rtfMRI-nf has clinical utility in reducing the symptoms of chronic pain[16], tinnitus[17], and Parkinson's disease[18]. Furthermore, a recent study in depressed men found the ability to up-regulate activity in various emotion-related brain regions though rtfMRI-nf was associated with clinical improvement[19]. It is important to note, however, that these clinical studies were pilot studies with relatively small samples (on the order of 5–12 patients) and replication and randomized clinical trials are needed to draw definitive conclusions regarding the clinical utility of neurofeedback procedures.

The current rtfMRI-nf study targets a brain region critically involved in both emotional processing and the pathophysiology of MDD: the left amygdala (LA). Studies show amygdala BOLD activity increases in response to both positive and negative emotional stimuli in healthy humans[20]–[22]. A functional dissociation between left and right amygdala has been proposed such that the right is engaged in rapid/automatic detection of emotional stimuli, while the left is involved in detailed and elaborate stimulus evaluation[21], [23]. While abundant evidence suggests LA hemodynamic responses to negative stimuli are exaggerated in MDD[24]–[26], extant evidence further suggests MDD-associated amygdala abnormalities are “doubly dissociated” from healthy individuals by virtue of showing a greater response to negative stimuli *and an attenuated response to positive stimuli* [20], [27]. Furthermore, amygdala responsiveness to positive stimuli is inversely correlated with depression severity[27], and this response increases following successful antidepressant pharmacotherapy[20] or Cognitive Control Therapy[28]. These findings suggest that altered amygdala activation to positive stimuli is clinically relevant and that some antidepressant drugs and cognitive therapies may exert their therapeutic effect by normalizing this emotional processing bias[29].

The current study aimed to determine whether depressed individuals are able to use rtfMRI-nf to enhance the amygdala hemodynamic response to positive autobiographical memories, and whether this ability alters mood. Specifically, we predicted MDD subjects receiving rtfMRI-nf regarding left amygdala activity would demonstrate greater activity in this region while contemplating positive autobiographical memories (AMs) compared to those who received rtfMRI-nf from a region putatively not involved in emotional processing. Furthermore, we predicted significant improvements in mood-ratings would be evident in the experimental relative to the control rtfMRI-nf group.

## Methods

### Subjects

The study was conducted at the Laureate Institute for Brain Research. The research protocol was approved by the Western Institutional Review Board. Human research in this study was conducted according to the principles expressed in Declaration of Helsinki. All subjects gave written informed consent to participate in the study and received financial compensation. Because it employed an experimental rather than clinical trial design, the study was not registered in a public trials database.

Twenty-three right-handed, unmedicated adults ages 18–55 who met the Diagnostic and Statistical Manual of Mental Disorders (DSM-IV-TR)[30]_ENREF_28 criteria for MDD in a current major depressive episode participated in the study. Volunteers, recruited from the community via advertisements, underwent screening evaluations at the Laureate Institute for Brain Research, including the Structural Clinical Interview for *DSM-IV* disorders[31]. Exclusion criteria included current pregnancy, general MRI exclusions, serious suicidal ideation, psychosis, major medical or neurological disorders, exposure to any medication likely to influence cerebral function or blood flow within three weeks (8 weeks for fluoxetine), and meeting *DSM-IV* criteria for drug/alcohol abuse within the previous one year or for alcohol/drug dependence (excepting nicotine) within the lifetime. All volunteers were naïve to rtfMRI neurofeedback.

### Experimental Paradigm

**Figure 1:**
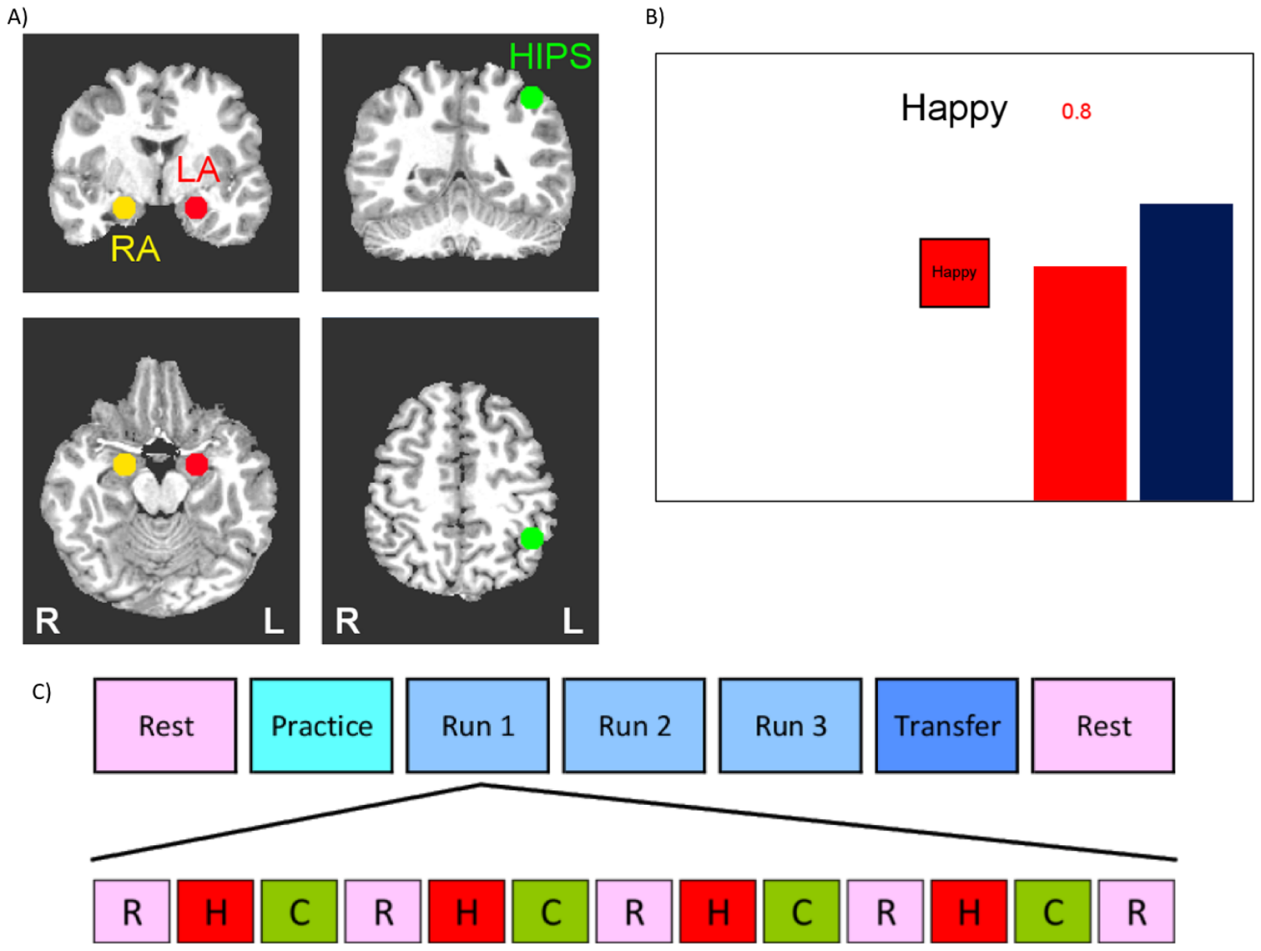
Design of the rtfMRI neurofeedback experiment. **A) Regions of Interest (ROI) for the rtfMRI neurofeedback procedure.** Three regions of interest (spheres of 7 mm radius) were used to assess changes in BOLD activity. These regions were the left amygdala (LA, red, centered at -21, -5, -16), right amygdala (RA, yellow, centered at 21, -5, -16), and left horizontal segment of the intraparietal sulcus (HIPS, green, centered at -42, -48, 48). ROI placements are illustrated on T1-weighted coronal (upper row) and axial (lower row) human brain sections in Talairach space[48]. Following radiological notation, the left side (L) of the brain is shown on the right, and the right side (R) of the brain on the left. **B) Real-time display screen for the rtfMRI neurofeedback procedure.** During the Happy condition, the word “Happy,” two color bars, and a number indicating the neurofeedback signal were displayed on the screen. Participants were instructed to recall happy autobiographical memories to make themselves feel happy while trying to increase the level of the red bar representing the feedback signal from the target ROI to a given target level indicated by the fixed height of the blue bar (but not to exceed that target level). **C) Protocol for the rtfMRI neurofeedback experiment.** The experimental protocol consisted of seven runs each lasting 8 min 40 sec. During the Rest runs, participants were instructed to rest with their eyes open. During the Practice run, the participants were given the opportunity to become comfortable with the procedure and test out different memories. During Runs 1–3 participants underwent rtfMRI neurofeedback training consisting of alternating blocs of Rest (R, pink block), Happy (H, red block), and Count (C, green block, instructed to count backwards from 300 by a given integer), each lasting 40 sec. During the Transfer Run, participants were instructed to perform the same task as during the neurofeedback training, but no neurofeedback information (bars, numbers) was provided.

The experimental paradigm is based on work previously published within our laboratory using healthy control subjects [14], and a task outline is depicted in Figure 1.

Participants were informed that they would be assigned to receive neurofeedback from one of two brain regions; one region involved in emotional processing or another region that is independent of emotional processing and which may be difficult to regulate. Participants were instructed to retrieve positive AMs that potentially would help them control the level of activity in the target brain region. The strategy of positive AM retrieval was selected based on findings of amygdala activity (with other medial temporal regions) during AM retrieval[32], and is commonly reported by participants post-hoc as an effective strategy in neurofeedback studies targeting emotional processing brain regions[12], [19], [33]. Because depressed individuals are impaired at recalling specific and positive AMs[34], [35], each participant was interviewed prior to scanning to facilitate their AM recall and ensure five highly arousing and vivid, specific, and happy AMs could be evoked during rtfMRI-nf. Participants were instructed to recall those or other happy AMs while attempting to increase the hemodynamic activity in the assigned ROI to that of the blue bar representing the target level of activation, but not to exceed that level. The decision to include the instruction not to exceed the target level was based on post-scan interviews with the first few participants who indicated that they tried so hard to get their amygdala level as high as possible during the Practice Run that they felt fatigued during the next training run. Therefore, we deemed the instruction not to exceed the target level necessary in order to avoid overexertion early in the task which could result in fatigue as the task progressed and the target level increased. They were informed to maintain this strategy even if they felt it was ineffective at raising their brain activity, though they could change the positive memories utilized or the aspects of the memories focused on.

Each neurofeedback run consisted of three conditions: Happy Memories, Count, and Rest. For each condition, cues were presented on the screen using both text and color icons to indicate each condition. During the Happy Memory Condition (Figure 1b), the cue “Happy” and two color bars (red, blue) were displayed on the screen. The red bar represented the actual neurofeedback signal, which was updated continuously by changing the height of the bar either upwards or downward based on the corresponding level of BOLD activity. This neurofeedback signal was also indicated by a number shown above the red bar representing the percent signal change within the target region. During this condition, participants were instructed to retrieve and contemplate positive autobiographical memories while also attempting to increase the level of the red bar to the fixed target level displayed by the blue bar. Because the Happy Memories condition required memory recall and rumination on those memories could potentially not be stopped quickly[13], [36], two control conditions were implemented to distract participants' attention from contemplating positive memories and to dampen the activation of the emotion regulation network[37]. During the Count condition, the participants were shown the cue “Count” with the specific instruction to count backwards from 300 by subtracting a specified integer. This integer was 3, 4, 6, 7, and 9 for Practice, Run 1, Run 2, Run 3, and the Transfer run respectively. During the Rest condition, participants were presented with the cue “Rest” and were asked to relax and breath regularly while looking at the display screen. No bars were displayed during the Count and Rest conditions.

The rtfMRI-nf procedure consisted of seven fMRI runs each lasting 8 minutes and 40 seconds (Fig 1c); a resting run, a practice run (PR), three training runs (R1-3), a final transfer run (TR) in which no neurofeedback information was provided, and a final resting run. During the Rest runs, a resting state paradigm was employed and participants were instructed to not think of anything in particular while fixating on the display screen. During the Practice run, participants were given an opportunity to become comfortable with the neurofeedback procedure. As with the training and transfer runs, the practice run consisted of alternating blocks of Rest (5 blocks lasting 40 seconds each), Count (4 blocks lasting 40 seconds each), and Happy (4 blocks lasting 40 seconds each). For the first three Happy Memory blocks participants were instructed to recall and contemplate the positive AMs prepared prior to the task with the experimenter, and then, for the last Happy condition block, to use the one memory that elevated their mood to the greatest extent. Thus, the Practice run allowed participants (i) to accommodate to the neurofeedback task, (ii) evaluate the emotional impact of the prepared happy memories within the experimental setting, and (iii) practice switching from one memory to another during neurofeedback training. During the subsequent 3 Training runs (Runs 1-3) the same alternating 40s blocks of Rest (5 blocks), Happy Memories (4 blocks), and Count (4 blocks) were presented. Participants were encouraged to try various other happy memories if the currently chosen one did not help them raise the red bar during neurofeedback training. Because our preliminary experiments indicated that the activation level of the left amygdala could be as high as a 2% BOLD signal change, the target level of the blue bar was set to 0.50%, 1.0%, 1.5% and 2.0% for PR, R1, R2, and R3, respectively. Finally, during the Transfer Run, the participants were instructed to perform the same task as during neurofeedback training, but rtfMRI-nf information was not provided for the Happy Memory blocks and the bars were not shown. The transfer run was performed to assess the transfer of the learned control and to check whether the training effect generalized to situations where no neurofeedback was available.

Prior to the rtfMRI-nf session, participants completed the 20-item Toronto Alexithymia Scale (TAS-20)[38], the Emotional Contagion Scale (EC)[39], and the Snaith-Hamilton Pleasure Scale (SHAPS)[40]. These ratings scales were selected as they examine the ability of individuals to experience emotion. As the current study trains participants to use the experience of internally generated positive emotions to regulate their amygdala, individual who have difficulty experiencing or describing positive emotions may have difficulty with the current neurofeedback task, and therefore ratings on these scales may explain important individual differences in the ability to perform the current task. Additionally, these scales were selected in order to compare our results to that of [14], which used the same neurofeedback paradigm in healthy control participants and found correlations between the ability to regulate the amygdala and ratings on these self-report measures. Clinician-administered rating scales included the 21-item Hamilton Depression Rating Scale (HDRS)[41], the Montgomery-Asberg Depression Rating Scale (MADRS)[42], and the Hamilton Anxiety Rating Scale (HARS)[43]. Both prior to and immediately following the fMRI session, participants completed the Profile of Mood States (POMS)[44], State/Trait Anxiety Scale (STAI)[45], and Visual Analog Scale (VAS). For the VAS, participants indicated along a 10 point scale (0 being not at all and 10 being extremely) how Happy, Sad, Restless, Angry, Anxious, Alert, and Drowsy they felt at the time of rating. The primary outcome measures for assessing the antidepressant effect were the POMS depression and VAS happy subscales because of their sensitivity to rapid changes in emotional state. Results obtained using other scales were considered secondary outcome measures.

Preliminary results of this study have been presented in abstract form at the Annual Meeting of the Organization for Human Brain Mapping[46], and the Annual Meeting of the American College of Neuropsychopharmacology[47].

### Region of Interest Placement

The rtfMRI-nf procedure was based on an MRI based region of interest (ROI) approach. Three ROIs were defined as spheres of 7 mm radius in the stereotaxic array of Talairach and Tournoux [48] and placed, respectively, in the left amygdala (LA: -21, -5, -16), the right amygdala (RA: 21, -5, -16) and the left horizontal segment of the intraparietal sulcus (HIPS: -42, -48, 48), as illustrated in Figure 1a. The neurofeedback signal was based on fMRI activation in the left amygdala ROI for participants in the experimental group and on the fMRI activation in the HIPS ROI for participants in the control group. Feedback was not given from the right amygdala ROI, rather this ROI was used in later analyses to determine laterality effects of our rtfMRI-nf procedure within the amygdala. The experimenter (KY) assigned participants on a 2∶1 ratio to either the experimental (LA rtfMRI-nf; n = 14) or control group (HIPS) rtfMRI-nf; n = 7) under double-blind conditions. The 2∶1 experimental/control ratio is commonly used in proof-of-concept rtfMRI-nf experiments[9], [12], [33]. Experimental procedures for both groups were identical, except control participants received rtfMRI-nf from a region putatively not involved in emotion regulation[49]–[51]. Upon completion of the study procedures, participants were informed as to which condition they were assigned, and participants in the HIPS rtfMRI-nf condition were offered the opportunity to return to the lab to repeat the rtfMRI-nf experiment with the amygdala as the target ROI.

The selection of a control task for rtfMRI-nf experiments is challenging, and no consensus has yet been reached as to the optimal approach. Studies utilizing out of scanner control conditions,[19] control conditions in which the neurofeedback bar remains static,[33] or no control condition (examining only within subject changes),[52], [53] run the substantial risk of false positives as control participants know they are not receiving feedback, and experimenter blinding is impossible. Therefore improvements evident in the active relative to the control group may be due to experimenter bias or the appeal of a novel, technology-based intervention and not to gaining control over the target region. Control conditions using neurofeedback from a different region are best suited to determine a) specificity of the procedure; whether feedback from the target region is necessary for enhanced control of that region and b) whether changes in mood ratings are due to feedback from the target region or due to a placebo effect. Therefore, for our rtfMRI-nf protocol, we employed a control condition in which subjects received rtfMRI-nf from the HIPS, a region implicated in number and not in emotional processing.[49]–[51], [54]

### Data Acquisition

MR imaging was conducted at the Laureate Institute for Brain Research using a General Electric Discovery MR750 whole-body 3 Tesla MRI scanner (GE Healthcare, USA) equipped with a custom rtfMRI system[55]. A standard 8-channel receive-only head coil array was used. A single-shot gradient-recalled EPI sequence with Sensitivity Encoding (SENSE) was employed for fMRI. The following EPI imaging parameters were used: FOV/slice  =  240/2.9 mm, axial slices per volume  =  34, acquisition matrix  =  96×96, repetition/echo time TR/TE  =  2000/30 ms, SENSE acceleration factor R  =  2 in the phase encoding (anterior-posterior) direction, flip angle  =  90°, sampling bandwidth  =  250 kHz, number of volumes  =  263. Each functional scan time lasted 8 min 40 sec. Three EPI volumes (6 sec) were added at the beginning of each fMRI run to allow the fMRI signal to reach steady state, and were excluded from data analysis. The EPI images were reconstructed into a 128×128 matrix, in which the resulting fMRI voxel volume was 1.875×1.875×2.9mm^3^. Additionally, simultaneous physiological pulse oximetry and respiration waveform recordings were conducted (with 50 Hz sampling) for each fMRI run. A photoplethysmograph with an infra-red emitter placed under the pad of the subject's left index finger was used for pulse oximetry, and a pneumatic respiration belt was used for respiration measurements. A T1-weighted magnetization-prepared rapid gradient-echo (MPRAGE) sequence with SENSE was used to provide an anatomical reference for the fMRI analysis. It had the following parameters: FOV  =  240 mm, axial slices per slab  =  128, slice thickness  =  1.2 mm, image matrix  =  256×256, TR/TE  =  5/1.9 ms, acceleration factor R  =  2, flip angle  =  10°, delay time TD  =  1400 ms, inversion time TI  =  725 ms, sampling band-width  =  31.2 kHz, scan time  =  4 min 58 sec.

### Imaging Analysis: On-line

The image data analyses were performed using Analysis of Functional NeuroImages (AFNI, http://afni.nimh.nih.gov/). The neurofeedback was implemented using the custom real- time fMRI system utilizing the real-time features of AFNI [56] and a custom developed graphic user interface (GUI) software. For each subject we acquired a high- resolution MPRAGE image and a short (10s) EPI scan prior to the neurofeedback procedure. The MPRAGE image was transformed to the Talairach space. The target ROIs (as defined above) were defined in Talairach space. They were first transformed to the original MPRAGE space, and then to the EPI space defined by a single EPI volume from the short EPI scan (for steady state). Thus, the target ROIs were defined in the EPI space. During the rtfMRI neurofeedback experiment, all acquired EPI volumes were volume-registered to the same single EPI volume. This way, the ROI masks in the EPI space were applied to all fMRI data in real time, and no Talairach transform during real-time processing was required.

The resulting ROIs in the EPI space contained approximately 140 voxels each. In our neurofeedback implementation, the AFNI real-time plug-in was used to perform volume registration of EPI images and to export mean values of fMRI signals for the three ROIs in real time. The first three volumes of each experimental run were excluded to allow the fMRI signal to reach steady state. The rtfMRI signal for each Happy Memories condition was measured as a percent signal change relative to the baseline obtained by averaging the fMRI signal for the preceding 40-sec long Rest condition block. This neurofeedback signal (percent signal change) was updated every 2 sec and displayed on the screen as the red bar. To reduce bar fluctuations due to noise in the fMRI signal, the bar height was computed at every time point as a moving average of the current and two preceding fMRI percent signal change values.

### Imaging Analysis: Post-scan Off-line

Pre-processing of single-subject fMRI data included correction of cardiorespiratory artifacts using AFNI implementation of the RETROICOR method [57]. The cardiac and respiratory waveforms recorded simultaneously during each fMRI run were used to generate the cardiac and respiratory phase time series for the RETROICOR. Further fMRI pre-processing included volume registration and slice timing correction for all EPI volumes in a given exam. Standard GLM analysis was then applied separately for each of the fMRI runs. The following regressors were included in the GLM model: two block stimulus conditions (Happy Memories, Count), six motion parameters as nuisance covariates to take into account possible artifacts caused by head motion, and five polynomial terms for modeling the baseline. The stimulus conditions for all runs consisted of 40-second-long blocks. Hemodynamic response amplitudes were estimated using the standard regressors, constructed by convolving a boxcar function (representing the block duration) with the canonical hemodynamic response function using standard AFNI parameters. The GLM β coefficients were computed for each voxel using the 3dDeconvolve AFNI program and then converted to percent signal changes for Happy versus Rest, Count versus Rest, and Happy versus Count contrasts. The resulting fMRI percent signal change maps for each run were spatially transformed to the stereotaxic array of Talairach and Tournoux [48] and re-sampled to 2×2×2 mm^3^ isotropic voxel size. They were subsequently used for whole-brain statistical group analyses. The voxel-wise percent signal change data were also averaged within the three ROIs (LA, RA, HIPS) and used as a performance measure. To obtain performance measures during the RE run, the GLM analysis procedure used for the neurofeedback runs (PR,R1,R2,R3,TR) was applied to the RE run. The same condition blocks (Happy, Count) were employed, even though there were obviously no such conditions during the resting run. We performed this procedure for additional verification of the results. The % BOLD activity levels for such condition blocks should be close to zero for RE runs, which was indeed the case in our analysis. In preparation for the whole-brain statistical group analysis, the spatially-normalized fMRI percent signal change maps were spatially smoothed using a Gaussian kernel with full width at half maximum (FWHM) of 5 mm.

For each group, statistical activation maps (t-tests comparing percent signal change to zero activation) were computed for Happy versus Rest, Count versus Rest, and Happy versus Count contrasts during TR. A group t-test comparing Happy versus Rest examined statistical differences between groups during TR. The significance criterion for detecting activation was set at *p*_corrected_<0.05 determined using the AFNI program 3dClustSim (cluster size>30 voxels, thresholded at voxel *p*<0.005). We expect activation detected during TR when no neurofeedback information was provided during happy AM recall reflects the regions involved in maintaining the elevated amygdala response to positive AM recall after completing rtfMRI-nf training.

### Behavioral Data Analysis

Analysis of behavioral data was performed using SYSTAT 13 (Systat Software Inc., USA). The training effect was evaluated by applying a three-way (Training [PR, R1, R2, R3, TR] x ROI [LA, RA, HIPS] x Group [Experimental, Control]) ANOVA for percent signal change. Specificity of the training effect to the LA was evaluated (within each group) by applying a two-way (Training x ROI) ANOVA for percent signal change. Follow-up t-tests were performed to characterize significant differences underlying ANOVA main effects and interactions. Monotonic properties of participants' control over brain activation across all runs were evaluated (for each ROI and Group) by using a one-way ANOVA trend analysis for repeated measures on percent signal changes with Training (RE, PR, R1, R2, R3) as a within-subjects factor. Association between average percent signal changes for LA ROI and scores on self-report measures was determined using Pearson bivariate correlations. The threshold for significance was set at *p*<0.05, one-tailed corrected for multiple comparisons using the Bonferroni method. A one-tailed test was selected for this proof-of-concept study because our aprioi hypotheses were directional in that we expected LA rtfMRI-nf to increase mood and amygdala BOLD activity.

## Results

### Baseline Demographic and Clinical Data

At the pre-treatment baseline evaluation (Table 1) the groups did not differ on mean age, HDRS, MADRS, or HARS scores, time since last antidepressant medication, or length of current depressive episode (ts(19)<0.84, ps>0.21). A Fisher's exact test revealed no significant difference between groups in the proportion of females, major depressive episodes experienced (1, 2, or 3+), or number of antidepressant medications previously taken (0, 1–2, or 3+) (*p*s>0.26). There was a difference between groups in the proportion of comorbid diagnoses (*p* = 0.045) such that the experimental group had more participants without a co-morbid diagnosis than the control group. When we compared pre-scan ratings (Table 2) between groups, there was no significant difference on any rating (ts(19)<1.61, *ps*>0.13).

**Table 1:** Participant Characteristics by Experimental Group.

	Experimental [n = 14]	Control [n = 7]
Age	38 (10)	36 (9)
% Female	79% [n = 11]	100% [n = 7]
Clinical Ratings – mean (SD)		
MADRS	27.1 (6.69)	31.4 (6.71)
HDRS	19.9 (5.15)	23.9 (5.49)
HARS	19.1 (5.32)	23.3 (7.74)
Mean length in years (SD) of current MDE	4 (5)	5 (5)
Number of Previous MDEs		
1 Episode	14.3% [n = 2]	14.3% [n = 1]
2 Episodes	21.4% [n = 3]	0.00% [0]
3+ Episodes	64.3% [n = 9]	85.7% [n = 6]
Comorbid Diagnoses*		
None	50.0% [n = 7]	0.00% [0]
GAD	14.3% [n = 2]	28.6% [n = 2]
Social Phobia	14.3% [n = 2]	42.9% [n = 3]
OCD	7.14% [n = 1]	0.00% [0]
PTSD	28.6% [n = 4]	57.1% [n = 4]
Past Antidepressant Use		
None	42.9% [n = 6]	14.3% [n = 1]
1-2	28.6% [n = 4]	42.9% [n = 3]
3+	28.6% [n = 4]	42.9% [n = 3]
Time (in years) since last antidepressant use	2.33 (2.53)	2.83 (1.83)

Numbers in () indicate one standard deviation of the mean. Numbers in [] indicate the number of participants in the reported category.

Abbreviations: GAD  =  generalized anxiety disorder; HARS  =  Hamilton Anxiety Rating Scale; HRSD  =  Hamilton Rating Scale for Depression; MADRS Montgomery-Asberg Depression Rating Scale; MDE  =  major depressive episode; OCD  =  obsessive-compulsive disorder; PTSD  =  post-traumatic stress disorder.

* Some participants had more than one co-morbid diagnosis

**Table 2:** Pre-Scan Mood Ratings for each experimental group.

Pre-Scan Self-Ratings	Experimental [n = 14]	Control [n = 7]
SHAPS	29.7 (5.94)	31.6 (6.73)
TAS		
Identifying	18.6 (6.58)	21.0 (4.50)
Describing	14.4 (5.15)	17.5 (4.60)
Externally Orienting	18.2 (3.87)	22.0 (4.54)
Total	51.2 (13.1)	60.4 (11.1)
EC		
Positive	3.32 (0.52)	3.12 (0.47)
Negative	3.32 (0.45)	3.30 (0.45)
Total	3.33 (0.31)	3.23 (0.33)
STAI		
State	44.3 (8.98)	43.1 (11.5)
Trait	55.4 (8.05)	57.0 (12.6)
POMS		
Depression	15.9 (9.05)	16.7 (13.7)
Tension	6.64 (6.72)	9.29 (9.25)
Anger	4.00 (5.16)	6.71 (10.3)
Vigor	9.00 (6.25)	6.29 (8.04)
Fatigue	13.8 (5.89)	11.1 (6.99)
Confused	6.71 (3.41)	7.86 (5.40)
Friendly	17.2 (7.12)	16.6 (5.03)
Total Mood Disturbance	20.1 (29.0)	28.9 (47.2)
VAS		
Happy	3.14 (1.70)	3.14 (2.11)
Restless	3.57 (1.74)	5.00 (3.32)
Sad	4.18 (2.80)	4.57 (2.99)
Anxious	3.54 (2.69)	4.57 (3.60)
Irritated	2.25 (2.75)	2.43 (2.64)
Drowsy	5.29 (2.87)	2.86 (2.91)
Alert	4.50 (2.77)	4.14 (2.61)

Numbers in parentheses indicate one standard deviation of the mean.

Abbreviations: EC  =  Emotional Contagion; POMS  =  Profile of Mood States; SHAPS  =  Snaith-Hamilton Pleasure Scale; STAI  =  State-Trait Anxiety Inventory; TAS  =  Toronto Alexithymia Scale; VAS  =  Visual Analogue Scale.

### ROI Analysis

Two participants (one/group) reported falling asleep during the task and their data was removed from the analysis, leaving a final sample size of 21.

**Figure 2:**
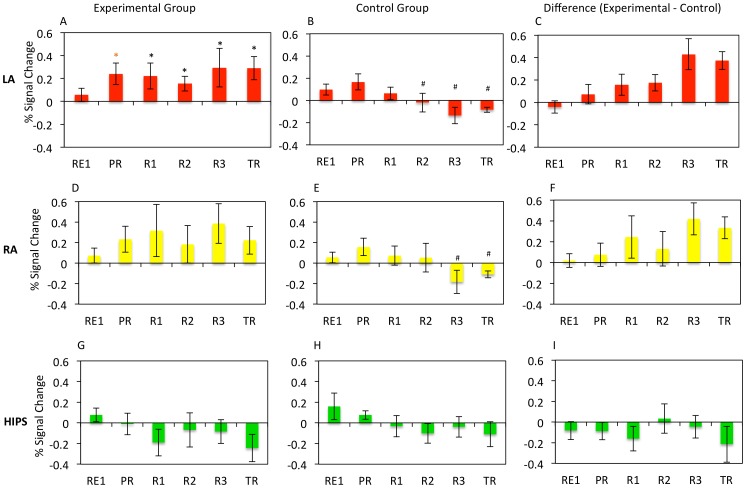
Percent BOLD Signal Change for each ROI, run, and group. Mean percent BOLD signal change for the Happy – Rest condition for each experimental run for the left amygdala (LA; panels A, B, C), right amygdala (RA; panels D, E, F), and horizontal segment of the intraparietal sulcus (HIPS; panels G, H, I) for the LA rtfMRI-nf group (panels A, D, G), HIPS rtfMRI-nf group (panels B, E, H), and for the difference between the LA and HIPS rtfMRI-nf groups (C, F, I). Error bars indicate +/− one standard error of the mean. * indicates a significant difference from 0 at p<0.05. * indicates a significant difference from 0 at p<0.10. # indicates a significant difference from the experimental group at p<0.05.

Results of the neurofeedback experiment based on the ROI analysis of BOLD data appear in Figure 2. A Training (PR, R1, R2, R3, TR) x ROI (LA, RA, HIPS) x Group (Experimental, Control) repeated measures ANOVA for Happy-Rest revealed a main effect of ROI (F(2,38) = 6.00, *p* = 0.005), an ROI x Group interaction (F(2,38 = 3.93, *p* = 0.02), and a significant ROI x Training x Group interaction (*F*(4,60) = 2.66, *p* = 0.045). These results indicate that the experimental and control groups differed in neurofeedback training effects on the BOLD signal based on the specific target region.

When the Training x ROI ANOVA was performed for groups separately, the experimental group had a significant effect of ROI (F(2,26) = 3.13, *p* = .025), but not Training (F(4,52) = 0.44, *p* = 0.39), and the ROI x Training interaction approached significance (F(4,52) = 2.24, *p* = 0.05). No significant main effects or interactions were found for the control group (Fs(4,24)<1.37, *p*s>0.14). This indicates the significant Training x Group and ROI x Group interactions were accounted for by effects on the BOLD signal in the target region of the experimental group only.

Follow-up t-tests examined significant ANOVA effects. Independent sample t-tests comparing percent signal change in each ROI for each run showed a significant difference between groups in the LA during Runs 2, 3, and TR (ts(19)>2.33, *p*s<0.038), while there was no significant difference between groups for HIPS BOLD activity during any run (ts(19)<0.97, *p*s>0.18). When examining the effects for the RA, there was a significant difference between groups during Run 3 (t(19) = 2.60, *p* = 0.01), and a during TR (t(19) = 2.02, *p* = 0.035) suggesting the effects of LA neurofeedback training, while specifically targeted to LA, also influenced right amygdala function.

One-sample t-tests (comparing activation to 0) assessed the significance of the BOLD change within each ROI and run. Within the LA in the experimental group there was a trend towards significance during PR (t(13) = 1.94, *p* = 0.05), and significant effects during R1, R2, R3 and TR (ts(13)>2.41, *p*s<0.02). No significant difference was found within the RA or HIPS for the experimental group (ts(13)<1.83, *ps*>0.024). In the control group, no significant difference was evident in any ROI or run (ts(6)<1.86, ps>0.08). These results suggest only participants in the experimental group were able to significantly elevate BOLD activity, and this effect largely was limited to the LA. Importantly, the finding that LA BOLD activity significantly increased during TR and did not differ from that in the final training run (TR v R3 (t(13) = 0.34, *p* = 0.37) in the experimental group suggests training effects persisted in the absence of neurofeedback.

Finally, we performed a one-way ANOVA trend analysis across all runs for LA activation. The experimental group showed a significant linear trend (F(1,19) = 4.68, p = 0.024) and the control group showed a nonsignificant trend (F(1,19) = 3.84, p = 0.05). These results were attributable to a linear *increase* in LA BOLD activity within the experimental group but a linear *decrease* in LA activity in the control group as training progressed (Figure 2). Tests for quadratic and cubic trends were not significant for either group (Fs(1,19)<0.80, *ps* >0.39). There was no significant change for either group within the RA (Fs(1,19)<2.36, *p*s>0.08) or HIPS regions (Fs(1,19)<0.75, *p*s>0.20).

### Associated Mood Effects

The mean changes on self-report emotional rating scales for each group are listed in Table 3. For the experimental group, significant changes were seen in our main outcome measures: POMS depression ratings decreased (t(13) = 2.12, *p* = 0.025), and VAS happiness ratings increased (t(13) = 3.04, *p* = 0.005) following LA rtfMRI-nf. The increase in happiness was significantly greater in the experimental compared to control group (t(19) = 2.33, *p* = 0.01), while the change in POMS depression between groups failed to reach significance (t(19) = 1.33, *p* = 0.10). Significant decreases were also found within the experimental group for ratings of POMS anger (t(13) = 2.42, *p* = 0.015), trait anxiety (STAI-Trait subscale; t(13) = 2.62, *p* = 0.01), state anxiety (STAI-Trait subscale; t(13) = 2.34, *p* = 0.02), and VAS ratings of restlessness (t(13) = 2.23, *p* = 0.02), anxiety (t(13) = 4.37, *p*<0.001), and irritability (t(13) = 2.14, *p* = 0.025), and the difference between the groups on state anxiety was significant t(19) = 2.58, *p* = 0.03). No other rating significantly changed pre-post scan (ts(13)1.85, *ps*>0.08). In the control group, VAS ratings of sadness decreased (t(6) = 2.57, *p* = 0.02), while no other rating score changed significantly pre-post scan (ts(6)<1.73, *p*s>0.10).

**Table 3:** Change in mood ratings (post-pre scan scores) following neurofeedback training for each experimental group.

Change Post-Scan	Experimental [n = 14]	Control [n = 7]
STAI		
State	−2.14 (3.21)*	3.86 (7.65)**
Trait^a^	−3.00 (4.30)*	−1.14 (3.39)
POMS		
Depression	−4.50 (8.48)*	−2.43 (5.26)
Tension	−3.00 (6.06)	−1.43 (5.86)
Anger	−2.36 (3.65)*	−3.29 (5.82)
Vigor	−0.29 (5.14)	0.29 (8.48)
Fatigue	0.57 (8.50)	−0.43 (5.32)
Confused	−1.00 (3.98)	1.29 (4.23)
Friendly	0.57 (6.32)	−0.14 (5.58)
Total Mood Disturbance	−9.21 (27.2)	−5.43 (29.0)
VAS		
Happy	1.75 (1.16)*	0.29 (1.63)**
Restless	−1.04 (1.73)*	−1.00 (3.37)
Sad	−0.93 (2.09)	−1.57 (1.62)*
Anxious	−1.79 (1.53)*	−0.57 (2.64)
Irritated	−1.36 (2.37)*	−0.86 (1.07)
Drowsy	0.11 (2,34)	0.72 (2.69)
Alert	−0.54 (2.53)	−0.86 (2.12)

Numbers in parentheses indicate one standard deviation of the mean. * indicates a significant change from pre to post-scan ratings at p<0.05. ** indicates a significant differences from the experimental group at p<0.05.

Abbreviations: POMS  =  Profile of Mood States; STAI  =  State-Trait Anxiety Inventory; VAS  =  Visual Analogue Scale

a One may wonder why the trait subscale of the STAI was administered both pre and post neurofeedback. While the Trait measure of anxiety is supposed to be a relatively stable measure, there is no set time period that the rating scale covers (such as the SHAPS which asks over the past week how often have you felt…) and instead asks how one “generally feels.” We wondered whether the neurofeedback procedure would alter how participants viewed themselves more generally, and indeed participants in the experimental group reported less general anxiety following the neurofeedback procedure. This could indicate they reflect more positively on themselves after the procedure and is another example of amygdala neurofeedback improving mood. However, as the test-retest reliability of the Trait measure of the STAI ranges from.65-.8645 (Spielberger CD, Gorsuch RL, R.E L (1970) Manual for the State-Trait Anxiety Inventory. Palo Alto, CA: Consulting Psychologists Press) the changes could simply be chance fluctuations in scores and replication is needed to confirm our hypothesis that how anxious one generally feels is indeed decreasing following amygdala neurofeedback

**Figure 3:**
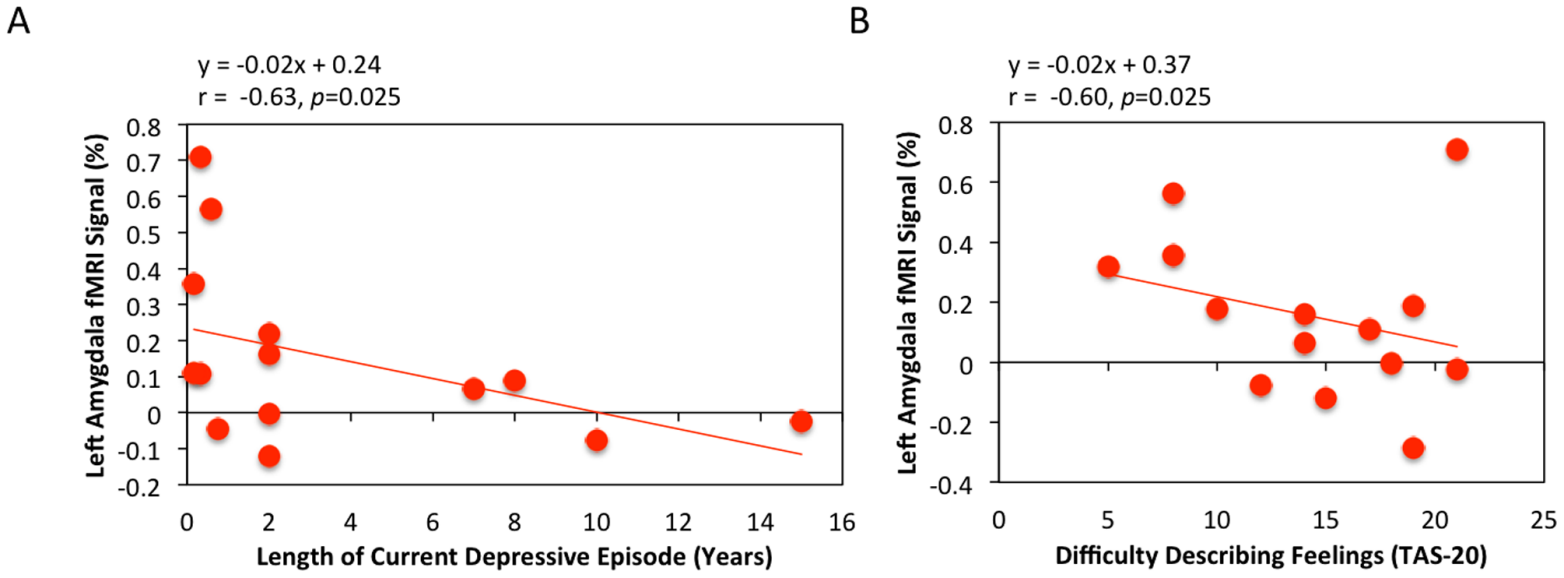
Relationship between left amygdala neurofeedback training effect and individual characteristics in the experimental rtfMRI-nf group. A) Correlation with length of the current major depressive episode. The training effect for the left amygdala (the average Happy – Rest percent signal change over the three training runs) was inversely correlated with the length of participants' current depressive episode. B) Correlation with difficulty describing feelings (TAS-20). The more difficulty a participant had describing their own feelings, the less average BOLD activation for the Happy-Rest contrast was observed in the left amygdala for the three training runs.

Finally, we tested whether there was an association between neurofeedback performance and clinical or mood rating variables. Within the experimental group, the mean percent signal change in the LA over the three training runs was inversely correlated with both the current depressive episode duration (r = −0.63, *p* = 0.025; Figure 3a), and the TAS “difficulty describing feelings” score (r = −0.60, *p* = 0.025; Figure 3b). No outliers were present in the data (see Figure S1 in Supporting Information). While the TAS scores followed a normal distribution (Shapiro-Wilk *W = *0.952, p = 0.563), the average MDE length did not (Shapiro-Wilk *W* = 0.739,p = 0.001). Therefore, we performed an additional correlation analysis on this data using Spearman's Rho and still found a significant correlation (ρ = −0.51, p<0.05) No significant correlations were found in the control group.

### Whole Brain Analysis

**Figure 4:**
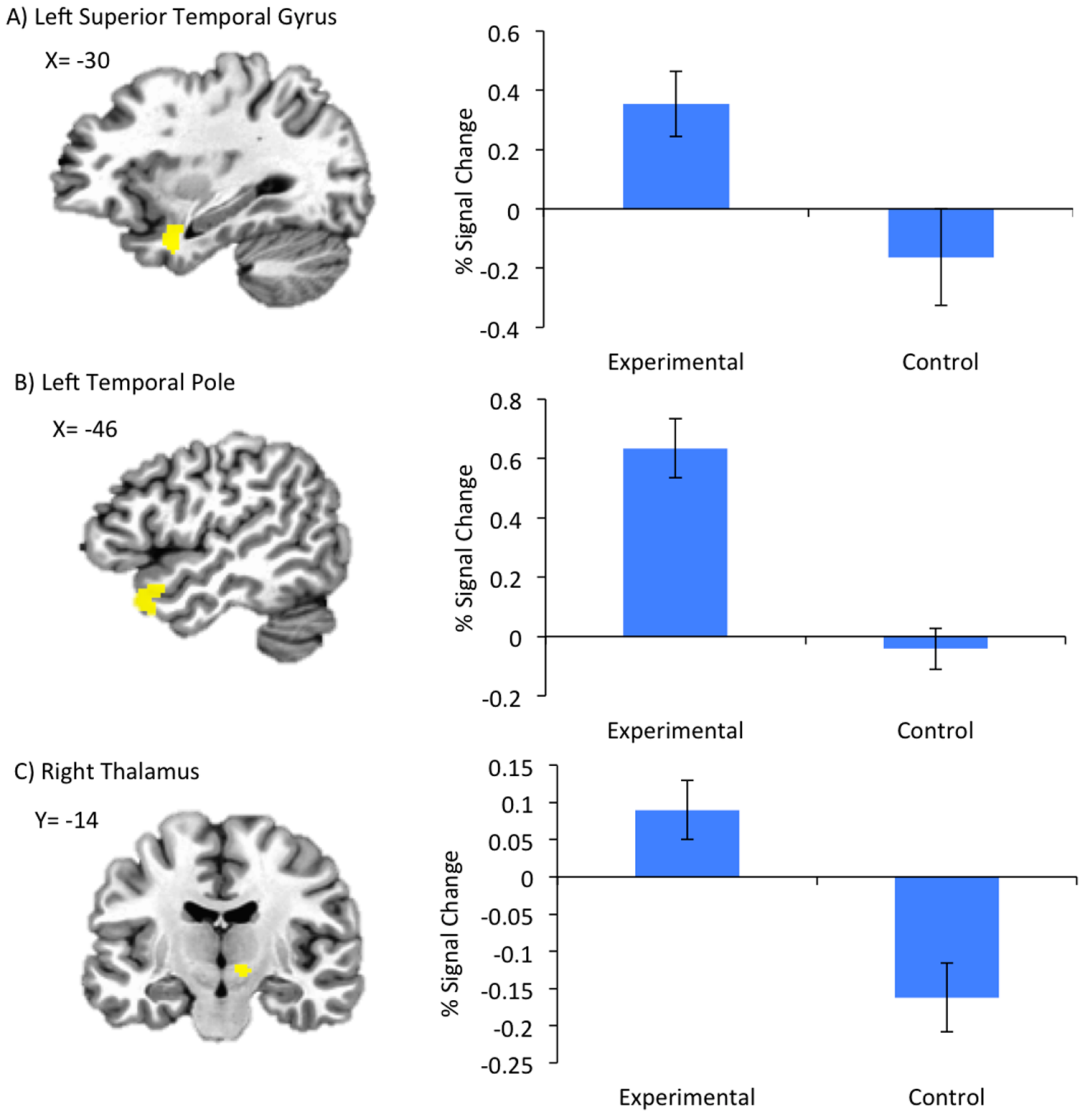
Group differences in BOLD activity during the Transfer Run. Regions A) Left Superior Temporal Gyrus B) Left Temporal Pole C) Right Thalamus and associated percent signal change graphs, are shown where groups had differential activation during Happy AM recall vs Rest during the Transfer run in which no neurofeedback was provided. Error bars indicate +/1 one standard deviation of the mean.

Results of fMRI data analysis for TR appear in Table 4. For the Happy versus Count contrast, the experimental group showed increased BOLD activity during positive AM recall within bilateral orbitofrontal cortex, dorsomedial prefrontal cortex, superior temporal and middle temporal gyrus, left ACC, posterior cingulate cortex, parahippocampal gyrus, and amygdala (encompassing the amygdala ROI) and right VLPFC and thalamus. The experimental group had increased activity during counting compared to positive AM recall in bilateral middle frontal gyrus, and inferior parietal lobe. When we compared regional BOLD activity between the Happy versus Rest conditions between the experimental and control groups during TR, the experimental group had increased activity in left superior temporal gyrus and temporal pole, and right thalamus (Figure 4).

**Table 4:** Regional increases in BOLD activity during the Transfer Run.

Area	Cluster Size	x, y, z	t score
**Happy AM condition > Count**			
L Orbitofrontal C	429	−37, 25, −10	8.16
R Orbitofrontal C	43	43, 23, 6	5.27
L DMPFC	581	−5, 51, 16	6.40
R DMPFC	41	5, 61, 22	5.56
R VLPFC	182	47, 23, 8	5.71
L Anterior Cingulate C	56	−5, 53, −2	5.39
L Posterior Cingulate C	850	−5, −53, 12	7.73
L Superior Temporal G	52	−43, −59, 18	4.76
R Superior Temporal G	371	37, 21, −22	6.16
L Middle Temporal G	167	−41, −5, −26	6.17
R Middle Temporal G	230	43, −57, −2	5.34
L Parahippocampal G	227	−25, −31, −14	5.32
L Amygdala	30	−23, −5, −15	5.17
L Thalamus	74	−3, −21. 10	5.23
**Count > Happy Memories**			
L Middle Frontal G	101	−23, −5, 52	5.09
R Middle Frontal G	69	43, 37, 26	4.55
L Inferior Parietal Lobe	769	−45, −39, 38	7.49
R Inferior Parietal Lobe	971	45, −57, 28	6.55
**Experimental > Control**			
L Superior Temporal G	140	−29, 3, −34	5.25
L Temporal Pole	71	−47, 17, −22	4.73
R Thalamus	31	11, −15, −4	5.24
**Control > Experimental**			
no significant clusters			

Coordinates correspond to the stereotaxic array of Talairach & Tournoux[48]. Cluster size refers to the number of contiguous voxels for which the voxel t value corresponds to *p*_corrected_<0.05.

Abbreviations: C  =  cortex; DMPFC  =  dorsomedial prefrontal cortex; G  =  gyrus; L  =  left; R  =  right; VLPFC  =  ventrolateral prefrontal cortex.

## Discussion

We investigated the feasibility of training MDD patients to regulate the hemodynamic activity their left amygdala using rtfMRI-nf and recall of positive AMs. Results demonstrate that, given appropriate direction, practice, and rtfMRI-nf information, MDD patients can enhance their amygdala BOLD activity by contemplating positive AMs within a single training session. That activity within the HIPS control region did not change over the course of the study in the experimental group suggests that feedback from the target amygdala region is necessary for enhanced control of that region and does not affect other regions not involved in emotional processing. Importantly, engaging in a single session of amygdala rtfMRI-nf (but not HIPS) training significantly improved mood. While the mental strategy of recalling positive AMs likely played a considerable part in this improved mood, neurofeedback from LA was crucial as evinced by lack of mood improvement in the control group who also recalled positive AMs while receiving rtfMRI-nf from the HIPS. Previous functional neuroimaging studies had implicated the HIPS region primarily in number processing[49]–[51], [54], along with visual attention[58], [59], and perceptual motor coordination[60], but not in emotional processing.

Although the right amygdala showed a nominal increase in the experimental group and decrease in the control group across the neurofeedback trials, this effect was only significant within the last training run of the experimental group. Statistically significant self-regulation was mostly specific to the target ROI of the LA. These data appear compatible with the lack of correlation between left and right amygdala glucose metabolism[61]. Nevertheless, the left and right amygdala share direct intrahemispheric anatomical connections[62], [63], and our data suggest the hemodynamic responses to our experimental task are not completely independent.

A linear trend was evident in which LA BOLD activity increased across runs in the experimental group, and decreased across runs within the control group. This suggests that patients in the experimental group were able to increase their LA BOLD response to positive stimuli, and to maintain this elevated response throughout the entire training session as well as during the transfer run in which neurofeedback was not provided. In the control group, amygdala BOLD activity was reduced over time, with no training effect evident in the HIPS. This decreased recruitment of the left amygdala over time in the control group could be related to the inability of MDD patients to maintain positive affect over time. It is well established that MDD patients suffer from anhedonia and an inability to sustain positive affect[64], [65], including an inability to sustain activity in brain regions involved in positive affect and reward[66]. That the experimental group was able to maintain this amygdala BOLD response during positive AM recall over the course of the training runs and into the transfer run further supports the clinical potential of the amygdala rtfMRI-nf procedure. While “Transfer” in the context of the current study refers to the ability to maintain the skill without feedback, it can also refer to the ability to perform the behavior within a different setting[67]. An important next step for future studies will be to examine whether training can be transferred to contexts outside of the scanner in order to further assess the clinical potential of the procedure.

An alternate explanation for the linear decrease evident in the HIPS rtfMRI-nf group is that receiving rtfMRI-nf from the HIPS could have influenced participants to change their mental strategy, which could have resulted in the decreasing amygdala activation. We find this an unlikely explanation as participants were explicitly instructed not to change their mental strategy and debriefing interviews suggest they followed these instructions. Furthermore, participants were not successful in regulating their HIPS activity (percent signal change did not differ from 0 in any run), again suggesting participants maintained the strategy of positive AM recall.

Notably, while both groups improved nominally on many of the self-report measures, only participants in the experimental rtfMRI-nf group showed statistically significant improvements. Participants in the amygdala rtfMRI-nf group reported significant decreases in ratings of depression, anxiety, anger, restlessness, and irritability along with significant increases in ratings of happiness, while mood ratings in the control group did not significantly change (except for a decrease in VAS sadness, conceivably due to the effects of positive AM recall). While it is conceivable that increasing the sample size in the control group would result in pre-post neurofeedback changes becoming significant, our conclusion that amygdala rtfMRI-nf improved mood is not solely based on significant within-subject changes in the experimental group and non-significant changes in the control group, but also significant improvements in anxiety and happiness ratings in the experimental relative to the control group. These results suggest that the experience of increasing LA BOLD activity during positive AM recall has therapeutic potential, beyond that of simply recalling positive AMs. While the control group did not have the experience of successful self-regulation associated with their AM recall, there was no evidence of any mood deterioration in these participants. Indeed, self-ratings of tension, anger, and irritability showed a nominal decrease in the HIPS rtfMRI-nf group that failed to reach statistical significance. Therefore, the mood effects observed in the amygdala rtfMRI-nf group are unlikely attributable to false positives due to increased frustration weakening mood effects in the HIPS rtfMRI-nf group. However, replication with a different control task or region is warranted to verify these results of improved mood in the experimental relative to control group.

We believe that the results support the conclusion that LA rtfMRI-nf resulted in the improved mood, and were not due to a placebo effect. The strategy of recalling positive AMs was not effective at increasing HIPS activation (evinced by no change from 0 in HIPS activity for any run in either group), therefore, one might expect participants to become unblinded as activation of the ROI was unresponsive to strategies of recalling positive AMs. We do not believe this to be the case, however, as during debriefing interviews in which condition assignment was revealed most participants in the experimental group expressed surprise and stated that they had believed themselves to be in the control HIPS condition. While surprising at first that even patients whose average LA activity was well above 0 would think they were in the control group, we believe this finding to be related to the negative self-schemas inherent to MDD [68]. That despite performing well, participants felt they must have been performing poorly. These findings argue against a placebo effect. Furthermore, these results suggest that amygdala rtfMRI-nf can result in mood improvements without conscious knowledge of training having occurred.

Not every participant in the experimental group was able to maintain an elevated amygdala response. The average LA BOLD response across the training runs (Runs 1–3) varied from +0.60% to −0.17%, and 4 participants in the experimental group had negative or near 0 amygdala BOLD responses indicating a failure to learn to regulate their amygdala. We found the amygdala training effect inversely correlated with length of the current depressive episode and with difficulty describing feelings. The correlation with depressive episode length suggests the clinical success of rtfMRI-nf of the amygdala may be dependent upon targeting patients early in the course of their depressive episode. This is consistent with previous research reporting that patients within a year of onset of their current depressive episode are more likely to respond to treatment than those whose episodes were of longer duration[69]. Indeed, patients in the current study who were within the first two years of onset of their current depressive episode were more likely to be able to regulate their amygdala activity via rtfMRI-nf.

The negative correlation between LA modulation and difficulty describing feelings is similar to that found in healthy controls engaging in the same paradigm[14]. However, an inverse correlation between amygdala modulation and difficulty *identifying* feelings (on the TAS) was reported within the healthy sample, while the current study found the correlation to exist within the *describing* feelings TAS subscale. These two TAS subscales are positively correlated and may load on a common factor related to emotional insight[70]. The observed correlation between insight and ability to regulate suggests certain MDD patients may be better suited to rtfMRI-nf treatment, as is the case for other psychological treatments for depression [71], [72].

Our results are partially consistent with those of the only study to date to examine the clinical potential of rtfMRI-nf training in MDD[19]. This work reported reduced depressive symptoms following a different rtfMRI-nf procedure, however, only clinician-administered ratings of depression improved following neurofeedback, and only following multiple sessions. In contrast, we found immediate improvements in self-reported mood ratings following a single neurofeedback session. The results of [19] must be interpreted with caution, however, as the control condition was performed outside of the fMRI environment, raising the question as to whether clinical improvement following neurofeedback were in fact due to learning to up-regulate brain regions involved with positive emotions or instead due to the appeal of a novel, technology-based intervention. Indeed, group differences in self-report measures (such as POMS) did not differ between experimental and control groups while non-blinded clinician administered ratings differed significantly, further suggesting that experimenter bias and placebo effects may have driven their results. Furthermore, the target neurofeedback regions used in [19] were adjusted, and could change, for each participant and run. While patients were able to increase BOLD activity in the target regions over time, interpretation and replication of these results is difficult as the BOLD response from run-to-run could include activity from several different regions. These different and changing regions makes it difficult to conclude that symptom improvement was not due to the general experience of gaining control over brain activity rather than gaining control over the specific regions modulated. The results from the current rtfMRI-nf protocol, in which the target region selection for neurofeedback was based on existing knowledge of emotional processing and psychiatric disorders, allows for easier interpretation of results and application for treatment designs.

The whole-brain voxel-wise analysis showed the ability to maintain elevated amygdala activity during positive AM recall following rtfMRI-nf training engaged a prefrontal-temporal cortical-limbic network implicated in emotion processing and AM recall[73], [74]. Many of these regions share extensive anatomical and functional connections with the amygdala and are recruited during emotional learning[73] and in the modulation of emotional processes[75]. These regions also form part of the core network recruited during AM recall[74], and showed increased hemodynamic activity in healthy males as they performed the same task[14], as well as increased functional connectivity following neurofeedback using the same procedure[76]. This pattern suggests that rtfMRI-nf from the amygdala is not dependent on a signal brain region, but upon a network. Future studies investigating rtfMRI-nf may benefit from using a network as opposed to a signal ROI.

The superior temporal gyrus, temporal pole and thalamus were more active in the experimental group than the control group during TR, indicating these regions are critically involved in maintaining the elevated amygdala response to positive AMs following training but in the absence of neurofeedback. Increased thalamic activity may reflect increased processing or relay of information between frontal and limbic regions recruited during the task[77], or increased arousal[78], which may be needed to maintain an elevated amygdala response during AM recall. Superior temporal and temporopolar regions are involved in emotional processing and social cognition[79]–[81], and are less active in MDD versus healthy individuals[82]–[84]. Therefore, the increased activity in these regions in MDD patients during LA but not HIPS rtfMRI-nf suggests the rtfMRI-nf procedure effectively recruits other regions important in emotional regulation which show abnormal BOLD responses in MDD, further suggesting potential for rtfMRI-nf in MDD treatment.

Some limitations of our study design merit comment. In this proof-of-concept study, the relatively small sample size, unmatched clinical characteristic of the experimental and control groups, and few male participants may limit the generalizability of our findings. While the control group appeared nominally more depressed than the experimental group, no differences reached significance with regard to clinical characteristics (except comorbidity), and ratings on the HDRS and MADRS for both groups were in the severely depressed range, further indicating comparable symptom severity. Nevertheless, future studies should include larger, more diverse, and better matched samples of MDD participants, which will enable better characterization of populations this treatment might best be suited to. Additionally, there was a relatively large inter-participant variability of the results, as mentioned above. While differences in the length of current depressive episode and ability to describe feelings partly accounted for this variability, other individual factors (e.g., learning ability, attention, motivation, fatigue) likely contributed as well. Repeating the rtfMRI-nf session multiple times would likely increase the training effect. Additionally, other means of enhancing training such as adjusting the target level of activation based upon individual performance should be considered in future studies. Furthermore, the current study included a single rtfMRI-nf training session, therefore we were unable to determine whether effects on clinician administered ratings (which usually cover a several day period) were evident or how long observed improvements on self-report mood ratings last, allowing us to conclude only that the amygdala neurofeedback procedure has short term benefits on mood. Additional sessions and ratings will allow us to further determine the potential of this rtfMRI-nf paradigm as an MDD intervention. Finally, randomization was not employed in the current study. Randomized blinded clinical trials are now needed to determine whether rtfMRI-nf might become a useful addition to current therapies for depression.

### Conclusions

In conclusion, our study demonstrates that MDD patients can self-regulate their amygdala activation using positive AM recall while receiving rtfMRI-nf. In contrast to the HIPS rtfMRI-nf, the feedback provided from the left amygdala resulted in a significant BOLD signal increase during rtfMRI-nf training, which persisted during TR in which no neurofeedback was provided. This training resulted in significant improvements in short-term state-dependent mood ratings, including increased ratings of happiness and decreased ratings of depression and anxiety only in the experimental group. In the experimental group, the training effect was inversely correlated with the length of the current depressive episode as well as with difficulty describing feelings, suggesting important individual differences are related to the ability to regulate the amygdala in response to rtfMRI-nf training. Further investigating characteristics associated with successful amygdala modulation and rtfMRI-nf related improvements of mood, along with studies aimed at improving and extending the neurofeedback effects, could ultimately allow this rtfMRI-nf procedure to be translated into a non-invasive MDD treatment.

## Supporting Information

Figure S1 **Box plots for the experimental group for a) average amygdala percent signal change over the 3 training runs (R1–R3) b) length of the current major depressive episode and c) the difficulty describing feelings subscale of the Toronto Alexithymia Scale.**
(DOCX)
