# Supplementary material for: Real-Time fMRI Neurofeedback Training of Amygdala Activity in Patients with Major Depressive Disorder
Source: PLoS One. 2014 Feb 11;9(2):e88785. doi: 10.1371/journal.pone.0088785 (PMC3921228; doi:10.1371/journal.pone.0088785)

Supporting Information

**Real-time fMRI neurofeedback training of amygdala activity in patients with major depressive disorder**

Kymberly D. Young, Ph.D., Vadim Zotev, Ph.D., Raquel Phillips, B.S., Masaya Misaki, Ph.D., Han Yuan, Ph.D., Wayne C. Drevets, M.D., Jerzy Bodurka, Ph.D.

**Outliers**

We inspected the correlational data for outliers. Because of the small sample size, we wanted to rule out the possibility that outliers were driving the significant correlations. The significant correlations were between the average left amygdala percent signal change over the 3 training runs and the Difficulty Identifying Feelings subscale of the Toronto Alexithymia Scale as well as with the length of the current major depressive episode. Standard box plots were created (Figure S1a-c) with outliers defined as a data point 1.5 times above or below the interquartile range. As can be seen in Figure S1, no outliers were present in the data for the experimental group for any of the variables examined. We can therefore conclude that our correlations were not driven by outliers.

**Figure S1:** Box plots for the experimental group for a) average amygdala percent signal change over the 3 training runs (R1-R3) b) length of the current major depressive episode and c) the difficulty describing feelings subscale of the Toronto Alexithymia Scale.

a)


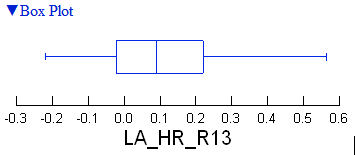


b)


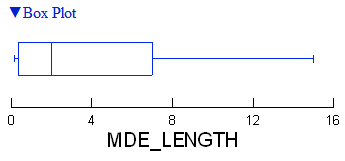


c)


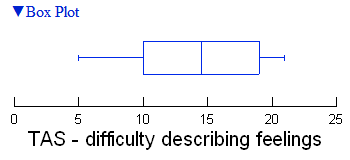

Supplement: Figure S1 — Box plots for the experimental group for a) average amygdala percent signal change over the 3 training runs (R1–R3) b) length of the current major depressive episode and c) the difficulty describing feelings subscale of the Toronto Alexithymia Scale. (DOCX) [file pone.0088785.s001.docx]
